# Cost-effectiveness of Artificial Intelligence-Aided Colonoscopy for Adenoma Detection in Colon Cancer Screening

**DOI:** 10.1093/jcag/gwad014

**Published:** 2023-04-07

**Authors:** Alan N Barkun, Daniel von Renteln, Hamid Sadri

**Affiliations:** Division of Gastroenterology, McGill University Health Center, Montreal, Quebec, Canada; Clinical Epidemiology, McGill University, Montreal, Quebec, Canada; Division of Gastroenterology, the University of Montreal Hospital and University of Montreal Hospital Research Center, Montreal, Quebec, Canada; Department of Health Economics and Outcomes Research, Medtronic Canada, Brampton, Ontario, Canada

**Keywords:** Adenoma detection, Artificial-intelligence, CADe, Colonoscopy, Colorectal cancer

## Abstract

**Background and Aims:**

Artificial intelligence-aided colonoscopy significantly improves adenoma detection. We assessed the cost-effectiveness of the GI Genius technology, an artificial intelligence-aided computer diagnosis for polyp detection (CADe), in improving colorectal cancer outcomes, adopting a Canadian health care perspective.

**Methods:**

A Markov model with 1-year cycles and a lifetime horizon was used to estimate incremental cost-effectiveness ratio comparing CADe to conventional colonoscopy polyp detection amongst patients with a positive faecal immunochemical test. Outcomes were life years (LYs) and quality-adjusted life years (QALY) gained. The analysis applied costs associated with health care resource utilization, including procedures and follow-ups, from a provincial payer’s perspective using 2022 Canadian dollars. Effectiveness and cost data were sourced from the literature and publicly available databases. Extensive probabilistic and deterministic sensitivity analyses were performed, assessing model robustness.

**Results:**

Life years and QALY gains for the CADe and conventional colonoscopy groups were 19.144 versus 19.125 and 17.137 versus 17.113, respectively. CADe and conventional colonoscopies’ overall per-case costs were $2990.74 and $3004.59, respectively. With a willingness-to-pay pre-set at $50,000/QALY, the incremental cost-effectiveness ratio was dominant for both outcomes, showing that CADe colonoscopy is cost-effective. Deterministic sensitivity analysis confirmed that the model was sensitive to the incidence risk ratio of adenoma per colonoscopy for large adenomas. Probabilistic sensitivity analysis showed that the CADe strategy was cost-effective in up to 73.4% of scenarios.

**Conclusion:**

The addition of CADe solution to colonoscopy is a dominant, cost-effective strategy when used in faecal immunochemical test-positive patients in a Canadian health care setting.

## Teaser

The use of computers in medicine is growing. Studies showed that computers increase the chance of finding cancer in colonoscopy. But the equipment is more expensive. We assessed the cost of using a computer for a colonoscopy. We showed that it is less costly and more successful than colonoscopy alone. We showed that using the computer for colonoscopy lowers the cost of Canada’ Health care.

**What you need to know**

Artificial intelligence is being increasingly used in Gastroenterology. Artificial intelligence and the application of a deep-learning computer-aided detection system on adenoma detection during colonoscopy or CADe technology in colonoscopy improves adenoma detection rates.

**What this study adds**

We assessed the cost-effectiveness of CADe colonoscopy in the Canadian publicly funded health care systems, showing it is less expensive and more effective than performing a colonoscopy alone when modelling for costs, life-years and quality-adjusted life-years gained over the lifetime of patients referred as a result of a positive faecal immunochemical test.

**How this study might affect research, practice or policy impact**

This study complements available evaluative information on the CADe colonoscopy technology, informing its appropriate diffusion to ­improve patient outcomes.

## BACKGROUND

The gold standard for the detection of precancerous colonic lesions is colonoscopy (1). However, lesions are still missed, accounting for 25% of interval colorectal cancers (CRC) (2). The current principal quality indicator for colonoscopy is an endoscopist’s adenoma detection rate (ADR) (3). Studies have shown that an increased ADR improves adjusted hazard ratios for interval CRCs and cancer-related mortality (4). Indeed, ADR directly correlates with outcomes, with a 1% increase in ADR resulting in a 3% decrease in CRC risk (5). Recent data have confirmed that the benefits of an increase in ADR apply to more contemporary examinations across many institutions and remain true after extensive adjustments for patient characteristics and procedural indications (6).

Over the past 5 years, there have been an emergence and proliferation of artificial intelligence (AI) clinical solutions targeting various therapeutic areas in Gastroenterology (7). These technologies often greatly rely on vast amounts of imaging information, which has been the case in the digestive endoscopy field, particularly colonoscopy (8). Clinical applications of AI to colonoscopy have included the detection of premalignant or malignant lesions, also known as computer-aided detection (CADe), as well as the characterization of such identified polyps (CADx) (9).

Although many CADe solutions are emerging, the first to be approved by the US Food and Drug Administration was the GI Genius solution (Medtronic PLC, Minneapolis, MN, the USA), with relevant available data having been published in the form of a recent multicentre, randomized clinical trial (RCT) and subsequent meta-analysis (8,10).

To complement such compelling efficacy evidence for polyp detection by AI-aided colonoscopy, the economic impact of CADe solutions must now be characterized to better determine the feasibility of adopting such technology in health care settings with limited resources, as is the case in the publicly funded Canadian health care system. The goal of this study was thus to evaluate the cost-effectiveness of AI-aided colonoscopy using a specific CADe solution in a Canadian health care setting for which high-quality clinical and generalizable data exist.

## METHODS

**Figure 1.**
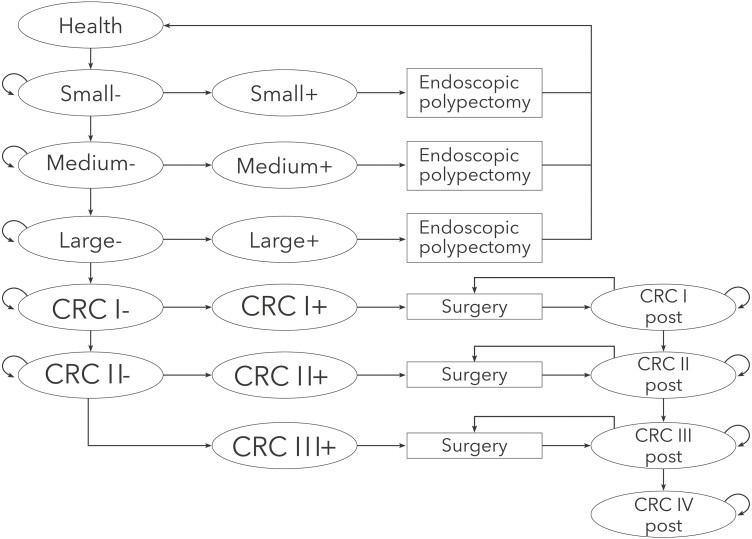
The Markov model with transition health states. CRC, colorectal cancer; roman numbers refer to the oncological staging.

We adopted a two-stage EXCEL-based (Microsoft Corp., Redmond, WA, USA) Markov simulation model with 1-year cycles and a lifetime horizon to estimate the incremental cost-effectiveness ratio (ICER) comparing CADe to conventional colonoscopy polyp detection rates (Figure 1).

### Patient Population, Index Colonoscopy Findings and Assumptions About Disease Progression

The target population was comprised of patients 50 years of age and older undergoing colonoscopy as a result of a positive faecal immunochemical test (FIT). We assessed this population since organized population-based CRC screening programs are principally FIT-based, as is the case throughout most of Canada (11). The prevalence of adenomas or CRC in the at-risk population was based on the recent Ontario colon cancer screening program report (12). Baseline adenomas or CRC diagnostic rates were derived from a recent meta-analysis as a function of adenoma size and CRC stage (8). Patient characteristics are displayed in Table 1.

**Table 1.** Baseline characteristics and disease state assumption

Parameter	Date			Source
Baseline cohort characteristics				
Starting age	50			Mittmann (2020) (13)
Sex (male)	54.5%			
Distribution among health states	FIT+	Screening	FIT+	Zorzi (2018) (14) Zorzi (2017) (15)
Healthy	40.43%	97.56%	40.43%	
Small adenoma (<5mm)	34.00%	1.39%	34.00%	
Medium adenoma (6–9 mm)	10.64%	0.44%	10.64%	
Large adenoma (>10 mm)	10.43%	0.43%	10.43%	
CRC stage I	2.37%	0.10%	2.37%	Zhao (2019) (16)
CRC stage II	0.90%	0.04%	0.90%	
CRC stage III	1.23%	0.05%	1.23%	
CRC stage IV	0.00%	0.00%	0.00%	

CRC, colorectal cancer; FIT, faecal immunochemical test.

### Adenoma Detection With and Without CADe and Missed Adenoma Rates

Assumptions for ADR in conventional and CADe colonoscopies were extracted from the pivotal RCT of 685 subjects undergoing a colonoscopy for broad indications, including initial screening with colonoscopy, post polypectomy, as a result of symptoms, or following a positive FIT (10). This sentinel trial demonstrated a significantly higher ADR amongst endoscopists assigned to use a real-time CADe when compared with conventional colonoscopy (54.8% vs. 40.4% - relative risk [RR], 1.30; 95% confidence interval [CI]: 1.14 to 1.45) (10).

The model is based on the adenoma missed rate (AMR) reported in the meta-analysis of specific for small, medium and large adenomas (16). For CRC stages I and II, the AMR of large adenomas is assumed to have reduced by 50%. We then calculated the probability of detecting adenomas or CRC correctly with colonoscopy (i.e., the sensitivity) as one minus the AMR.

The sensitivity of colonoscopy using the CADe solution was then estimated by applying the incidence rate ratio (IRR) of adenoma per colonoscopy (APC) reported by Repici (10). The risk reduction of CADe colonoscopy was calculated by multiplying the AMR by the IRR. The risk reduction of CADe colonoscopy was thus 15.5% for small adenomas (<5 mm), 8.28% for medium adenomas (6–9 mm) and 7.6% for large adenomas (>10 mm). For CRC, we used the per-patient detection rate’s RR as a proxy. All the assumptions relating to the polyp detection rates and incremental benefits provided by the CADe solution are listed in Table 2.

**Table 2.** Measures of polyp detection rates

Parameter	Data			Source
AI-aided detections rate	Rate			Hassan (2021) (8)
Small adenoma (<5 mm)	17.26%			
Medium adenoma (6–9 mm)	8.28%			
Large adenoma (>10 mm)	7.60%			
Incident rate ratios of initial or subsequent colonoscopy	Mean	95% CI		Repici (2020) (10)
Small	1.64	1.24	2.18	
medium	1.64	1.24	2.18	
large	1.07	0.66	1.74	
CRC	3.36	0.93	12.11	
Missed adenoma rates				Zhao (2019) (16)
Small adenoma (<5 mm)	31.00%	25.00%	38.00%	
Medium adenoma (6–9 mm)	19.00%	12.00%	28.00%	
Large adenoma (>10 mm)	9.00%	4.00%	16.00%	
CRC stage I	4.50%	—	—	
CRC stage II	4.50%	—	—	

AI, artificial intelligence; CI, confidence interval.

### Model Structure and Probability Assumptions

In the first stage of the model, patients were assessed for CRC in each cycle; the missed polyp can stay at the same stage or increase in size, which results in a subsequent endoscopic polypectomy at follow-up examination. As per CRC screening guidelines, patients with a negative colonoscopy (e.g., no adenoma or CRC at the index colonoscopy) were considered healthy and had a follow-up FIT after 10 years (17). Patients diagnosed with smaller adenomas (<10 mm or less than three polyps and no high-grade dysplasia) had a colonoscopy after 5 years, while high-risk patients (>10 mm or more than three polyps or exhibiting high-grade dysplasia) underwent a colonoscopy after 3 years; these were the recommended guidelines at the time the study was planned (18).

Amongst patients who would prove to have CRC, patients with large polyps entered the second stage of the model after subsequent assessment for disease progression. In each cycle, the polyp size could remain the same or increase and progress to the next CRC stage (CRC stages I–IV). Possible therapeutic interventions included endoscopic polypectomy, surgery, chemotherapy or radiotherapy (for advanced CRC) and follow-up care. All the assumptions relating to disease progression and health state transitions are listed in Table 3.

**Table 3.** Model input health state transitions

Parameter	Data			Source
Post-surgery CRC recurrent rate				Gilard-Pioc (2015) (19)
CRC stage I	5.8%			
CRC stage II	5.8%			
CRC stage III	18.8%			
CRC stage IV	18.8%			
CRC mortality rates	Age			
	<65	65–75	75+	Gilard-Pioc (2015) (19)
CRC stage I (no recurrence)	3.0%	5.0%	10.5%	
CRC stage II (no recurrence)	3.0%	5.0%	10.5%	
CRC stage III (no recurrence)	5.0%	8.5%	16.5%	
CRC stage IV (no recurrence)	5.0%	8.5%	16.5%	
CRC stage I (post recurrence)	56.0%	56.0%	87.0%	
CRC stage II (post recurrence)	56.0%	56.0%	87.0%	
CRC stage III (post recurrence)	68.0%	67.0%	93.5%	
CRC stage IV (post recurrence)	68.0%	67.0%	93.5%	
Health states transition probabilities	Mean	95% CI		Coretti (2020) (20)
Healthy to small adenoma (5 mm)				
Age 50	0.8%	0.4%	1.7%	
Age 55	1.0%	0.5%	2.0%	
Age 60	1.2%	0.6%	2.3%	
Age 65	1.3%	0.7%	2.7%	
Age 70	1.5%	0.8%	3.0%	
Small to medium (6–9 mm)	3.5%	1.7%	6.9%	
Medium to large (10 mm)	2.2%	1.1%	4.3%	
Large to CRC stage I	37.0%	26.8%	47.2%	
CRC stage I to stage II	23.8%	20.6%	27.1%	
CRC stage II to stage III	48.5%	32.1%	65.0%	
CRC stage III to stage IV	30.2%	15.1%	60.4%	

CRC, colorectal cancer; CI, confidence interval.

The probabilities of transitions between health states (in the Markov cycles) were calculated for the following parameters: utility scores, adenoma size (small, medium, large) and CRC progression (stages I–IV), adenoma missing rates (AMR), IRR per colonoscopy (Table 3).

### Effectiveness Assumptions

The outcomes of effectiveness were life years (LY) gained and quality-adjusted life years (QALY). LY and QALY are recognized and commonly used measures of effectiveness in economic studies (21). QALY is a single index generated by combining disease mortality and morbidity (expressed by the utility values in the cost-effectiveness studies) and is commonly used as a measurement tool for effectiveness and can assist health policymakers in setting priorities among competing health care technologies (22).

The mean utility value attributed to having an adenoma was 0.91 (95% CI: 0.87 to 0.93), and the mean utility value for CRC in various stages was: Stages I and II: 0.67, 95% CI: [0.62 to 0.72]; Stage III: 0.59, 95% CI: [0.54 to 0.69] and Stage IV: 0.25, 95% CI [0.20 to 0.31] (20).

### Health Care Resource Utilization and Associated Cost Assumptions

Direct medical costs associated with health care resource utilization were used in the analysis. The cost variables for various resources included FIT, colonoscopy, endoscopic polypectomy (for discovered and endoscopically resectable adenomas), surgical procedures for patients diagnosed with CRC stages I, II and III, adjuvant chemotherapy for CRC patients with surgical findings of stage III CRC, systemic chemotherapy for patients diagnosed with CRC stage IV, and follow-up visits (Table 4).

**Table 4.** Health care resource utilization and associated costs

Resources	Cost (+15%)	Source
FIT	$31.11 ($35.77)	Goede (2017) (23)
Colonoscopy	$688.00 ($791.20)	OCCI (2018) (24)
Endoscopic polypectomy	$413.00 ($474.95)	
CRC I–III surgical treatment	$12,082.00 ($13,894.30)	Springer (2019) (25) OCCI (2018) (CMG: C182, C184, C186, C187–189)
Adjuvant chemotherapy (CRC III)	$9,637.00 ($11,082)	Meyers (2015) (26)
Systemic chemotherapy (CRC IV)	$11,442.00 ($13,158.30)	Mittman (2020) (13) Paszat (2021) (27)
CRC stage I post-surgical follow-up	$306.00 ($351.90)	In the year post-surgery: colonoscopy, abdominal instrument exams every 6 months, laboratory exams every 3 months—OCCI 2018
CRC stages I and II post-surgical follow-up	$1,427.85 ($1,642.14)	In the year post-surgery: colonoscopy, abdominal instrument exams every 6 months, laboratory exams every 3 months, 1 PET— OCCI 2018

CMG, case-mix grouping; CRC, colorectal cancer; FIT, faecal immunochemical test; OCCI, Ontario case-costing initiative; PET, positron emission tomography.

According to the Canadian health economics guidelines, costs and outcomes were discounted yearly at 3.5% (28). All costs were expressed in 2022 Canadian dollars and were sourced from the published literature, the Ontario case costing Initiative, and the Canadian Institute for Health Information patient cost estimator (24,29). The costs of physician consultations were derived from the Ontario schedule of benefits: physician services (30). The cost of the CADe solution was estimated at $2,250 for a monthly subscription, as provided by the manufacturer (Medtronic Canada ULC, Brampton, ON, Canada). All cost assumptions were varied over a range extending to 15% beyond appropriate point estimates (Table 4).

This analysis compares conventional colonoscopy with CADe from a Canadian provincial payer perspective. The acceptable willingness-to-pay (WTP) value in Canada was set a priori at $50,000 per QALY.

### Sensitivity Analyses

We performed both deterministic and probabilistic sensitivity analyses using a Monte Carlo simulation on a hypothetical cohort of 1,000 patients on the main outcome variables to assess the robustness of the model. In particular, with regards to the confidence intervals around the difference in ADR point estimate comparing a colonoscopy performed with CADe versus not. The simulation of the point estimate of the difference in ADR was varied across the full IRR 95% confidence interval based on the published RCT to calculate the standard error. We adopted this approach as this represents the most conservative approach. However, as it is clinically unlikely that a colonoscopy performed with CADe solution would result in a lower ADR compared with conventional colonoscopy, we also completed the probabilistic sensitivity analysis varying the ADR difference only across the range of values over which polyp detection improved with CADe.

## RESULTS

### Incremental Cost-effectiveness Ratios

LYs gained in the CADe colonoscopy and conventional colonoscopy groups were 19.144 and 19.125 (*P* = 0.019), respectively. The QALY gains for CADe colonoscopy and conventional colonoscopy were 17.137 and 17.113 (*P* = 0.024) per thousand patients. The total 3-year acquisition cost of the CADe technology was calculated at $81,000.

If applied to 1,000 colonoscopies yearly, the per-case cost of CADe colonoscopy and conventional colonoscopy were $2,990.74 and $3,004.59, respectively, resulting in approximately $14 overall cost savings in the CADe group.

With a WTP threshold set at $50,000 per QALY, the ICER was the dominant strategy (offering better outcomes at a lower overall cost) for both outcomes, showing that performing colonoscopy using CADe solution is a cost-effective strategy in the Canadian health care system (Table 5).

**Table 5.** Cost-effectiveness of the artificial intelligence computer-aided diagnosis in polyp detection at colonoscopy

Parameter	Conventional colonoscopy	CADe colonoscopy	CADe vs. conventional colonoscopy	ICER (cost per LY)	ICER (cost per QALY)
LY	19.125	19.144	0.019	Dominant^*^	Dominant^*^
QALY	17.113	17.137	0.024		
Total cost	$3,004.59	$2,990.74	−$13.85		

^*^Dominant strategy means that the technology provides better outcomes at lower costs.

CADe, Artificial intelligence computer-aided diagnosis in polyp detection; ICER, incremental cost-effectiveness ratio; LY, life years; QALY, quality-adjusted life years.

### Sensitivity Analyses

**Figure 2.**
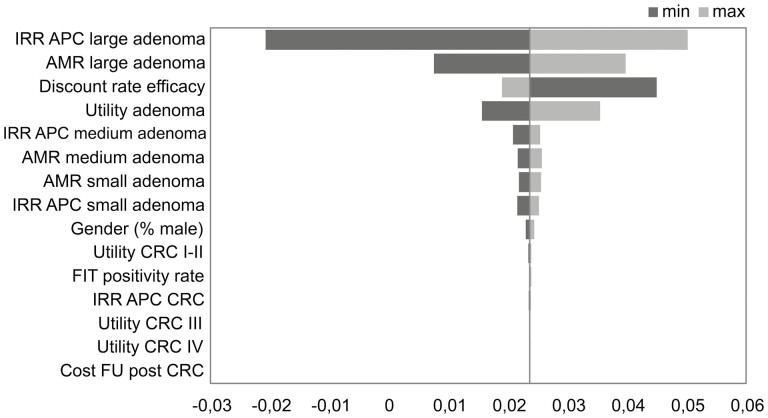
Deterministic sensitivity analysis when varying different effectiveness and cost assumptions. AMR, adenoma miss rate; APC, adenoma per colonoscopy; CRC, colorectal cancer; roman numbers refer to the oncological staging; FIT, faecal immunological test; IRR, incident rate ratio.

The deterministic sensitivity analysis demonstrated that the model was sensitive to between-group differences in the incidence risk ratio of adenoma and adenoma miss rates per colonoscopy for larger adenomas as main cost drivers and, to a lesser extent, assumption about adenoma utility values (Figure 2).

**Figure 3.**
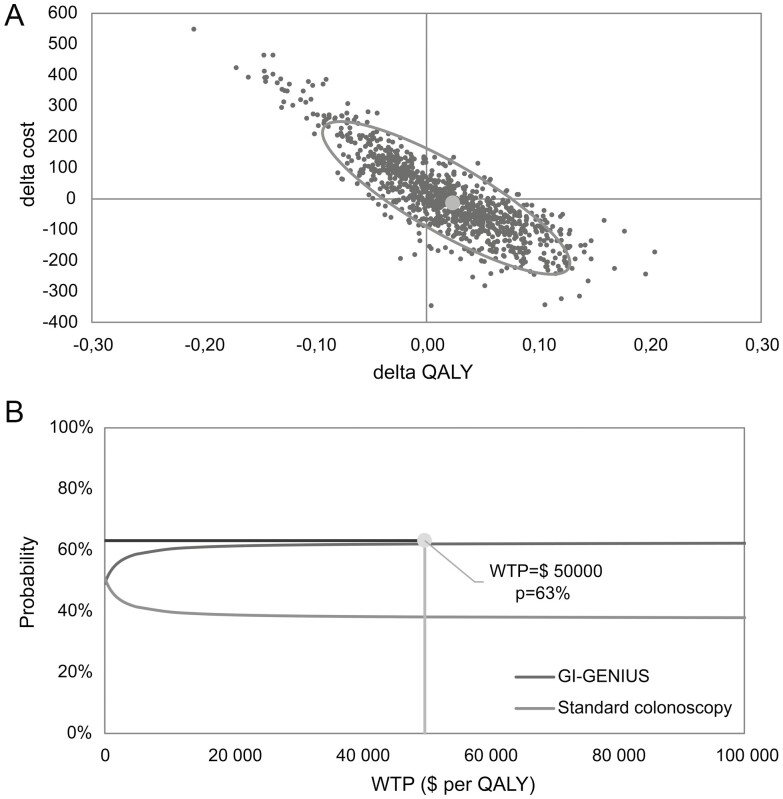
Cost-effectiveness graphs contrasting CADe with conventional colonoscopy. (A) Cost-effectiveness plane. (**B**) Cost-acceptability curve. *Delta in the *X* and *Y* axis refers to the difference in QALY and cost. **Probability that the strategy is cost-effective for a given simulation while varying cost and effectiveness assumptions. $, 2021 Canadian dollars; QALY; quality; WTP, willingness to pay.

The probabilistic sensitivity analysis showed that the CADe strategy was cost-effective in 63% of simulations when the difference in ADR across its entire 95% confidence interval was varied (Figure 3). This value rose to 73.4% of simulations when varying baseline assumptions only across an improvement in the ADR range attributable to the CADe solution (the latter is not shown in Figure 3).

## DISCUSSION

The rapid expansion of AI in health care highlights the importance of evaluating the economics of AI-aided technologies in policymaking. The current analysis is timely, as numerous trials and meta-analyses have shown the effectiveness of AI technology in colorectal adenoma detection (31,32).

This study demonstrates that the CADe solution is cost-effective compared to conventional colonoscopy amongst FIT-positive patients in the Canadian health care system, exhibiting a dominant ICER. The chosen study patient population is critical to organized CRC screening programs as most are based on FIT testing, as in Canada (11). Despite the upfront acquisition and maintenance costs of the AI technology, this analysis suggests that the overall cost per case would decrease if adopted, using a lifetime horizon amongst patients undergoing colonoscopy for a positive FIT. The plausible biological rationale is that the higher ADR results in a better diagnostic yield and treatment of precursor lesions before they become cancerous. Consequently, there is an improvement in the patient’s quality of life over a lifetime while decreasing overall health care costs, explained by a reduced number of downstream colon cancer treatments and follow-ups. The deterministic and probabilistic sensitivity analyses cover a wide range of effectiveness variables (e.g., IRR APC relative to the adenoma size) and costs (e.g., treatment and surgical procedures costs), confirming the robustness of our analysis, using both conservative and more liberal ranges of ADR differences between the CADe and conventional colonoscopy approaches. We selected data from the GI Genius CADe solution as it is one of the first commercialized, best-studied, and one of only a few approved CADe solutions in Canada and the United States. AMR estimates used in this analysis that were taken from a study by Zhao et al. overlap greatly with an additional recent report by Wallace et al. (33). A recent study also demonstrated a significant increase in ADR attributable to CADe, specifically in patients undergoing colonoscopy for a positive FIT; importantly, the ADR and its range overlap extensively with the assumptions of our model, supporting the validity and robustness of our findings (34). Interestingly, ADR estimates and ranges attributed to CADe from a large recent meta-analysis that included ten RCTs studying patients with a broad range of indications for colonoscopy (*n* = 6,629) while regrouping multiple CADe technologies overlapped completely with the assumptions used in our model (ADR = CADe 35.4% vs. standard 25.9%, RR = 1.43 [1.33–1.53]) (30). Importantly, a recent meta-analysis by Huang et al. has also confirmed improved total numbers of sessile serrated and advanced adenomas per colonoscopy, for which individual studies had yielded variable results due to smaller numbers of these rarer lesions (31).

The results are aligned with preliminary findings from recent international studies published as sole abstracts to date. These include Jootun et al., who used a Markov model simulation to evaluate the cost-effectiveness of CADe in a cohort of 1,000 patients aged 50 with a time horizon extending from screening and diagnosis through a patient’s life span (35). The measures of effectiveness included LYs gained, CRCs prevented and QALYs. The authors showed that the CADe strategy was cost-effective in the Spanish health care system, achieving an incremental 0.033 QALY gain with a WTP threshold set at €20,000—€30,000 per QALY (35). A comparable analysis from Italy yielded similar findings from an Italian national health care system perspective (36).

Areia et al. also reported that a CADe solution yielded greater QALYs at lesser costs with assumptions pertinent to a US setting in a secondary analysis of a Markov microsimulation that principally reported on colon cancer incidence and mortality as well as costs of implementing a CADe solution (37). They demonstrated that the CADe solution resulted in a 4.8% incremental gain for relative reduction of CRC incidence and a 3.6% incremental gain for CRC-related mortality. CADe thus decreased the cost per screened individual from USD 3,400 to USD 3,343 (a saving of USD 57 per individual). Similarly, they reported that a once-in-a-lifetime colonoscopy performed with CADe solution for the United States population would result in additional yearly prevention of 7,194 CRC cases and 2,089 related deaths at an annual saving of USD 290 million.

Although at the macro level, the full economic analysis that considers both cost and outcomes (e.g., cost-effectiveness) is a key decision determinant for policymakers to assess the feasibility of a technology, at a meso-level, understanding the budget impact on a system is helpful for financial planning (38). As such, a few additional studies have evaluated the cost consequences of AI-aided colonoscopy in other health care systems using different perspectives. Döring et al. assessed the cost impact of CADe on the German health care system by adopting data from a recently published meta-analysis on CADe (39). They concluded that a combination of CADe solution with polyp management could lead to cost control in German’s CRC screening program.

The economic benefits of AI may extend to polyp characterization and not just detection. Indeed, in an add-on analysis to their single-group, open-label, prospective clinical trial, Mori et al. estimated that the diagnose-and-leave strategy with AI-aided polyp characterization (CADx) could reduce the average colonoscopy cost and the gross annual reimbursement for colonoscopies by USD 149.2 million (18.9%) in Japan, USD 12.3 million (6.9%) in England, USD 1.1 million (7.6%) in Norway and USD 85.2 million (10.9%) in the United States, compared with a resect-all-polyps strategy as is most often current practice (40).

Indirectly addressing potential cost impacts, a retrospective analysis from the United States evaluated CADe solution and concluded that despite some concerns about potential negative effects on efficiency in high-volume ambulatory surgical centres, such CADe solution use was not associated with a significant increase in procedural time (41).

Comparable to other medical procedures that are dependent on operator judgment and decisions, colonoscopy outcomes (e.g., ADR) are quite operator-dependent and often relate to operator experience with better outcomes for patients who have access to centres with more experienced clinicians (42). Similar to other advanced technologies, AI-aided procedures have thus the potential to improve health equity by democratizing access to enhanced diagnostic results or treatment options (43). It is the case, more specifically, for CADe (and possibly CADx), as optimizing the diagnosis and outcomes of colonoscopy may lead to increased health care system efficiencies while also yielding potential cost savings (44). Adopting a societal perspective, the improved efficiency may help decision-takers finance additional advanced technologies for patient care in other therapeutic areas (45). Furthermore, like other AI technologies, CADe performance will likely further improve through ongoing annotated data inputs.

## LIMITATIONS

This study must be interpreted in the context of some limitations. We used a simulation economic model, which imposes methodological structural limitations. Indeed, there exists uncertainty around effectiveness outcomes reported in international publications. However, as discussed above, extensive sensitivity analyses addressed the uncertainty of the effectiveness and cost model assumptions. The study population focused exclusively on FIT-positive individuals, which represents a varying proportion of all patients undergoing a colonoscopy in an endoscopy unit—a proportion that is dependent on referral indications amidst the presence or absence of an organized CRC screening program. This study was conducted using the Ontario colon cancer screening program and cost data from a Canadian provincial health care perspective (17). Ontario has a well-established CRC screening program, and success rates may differ in other jurisdictions in Canada or internationally. Similarly, this analysis used a provincial payers’ perspective, including direct medical costs, and did not account for potential societal benefits and indirect and intangible costs, which would further enhance CADe-related benefits. As mentioned in the methods section, ADR assumption ranges included theoretical worsening polyp detection when using CADe, even if clinically unlikely, highlighting the conservative nature of the base-case analysis and its main conclusions. When only including the beneficial ‘positive interval’ of the full CADe-related ADR range difference, the proportion of cost-effective simulation increased from 63% to 73.4%. Additional unknowns relating to model assumptions include a possible levelling off of CRC benefits with greater ADR values (6), the impact of longer post-polypectomy screening intervals (18) (here, too, resulting in more conservative estimates in our analysis), and reliable estimates for AMR of larger lesions (33,46) that may in part be attributable to limitations in parallel versus tandem RCT colonoscopy study designs (47,48). Although this study adopted a Canadian payer’s perspective, due to the conservative nature of our assumptions and the higher WTP threshold usually adopted in the United States, it is likely that an effective CADe solution is good value for money and would be proven a cost-effective strategy in the United States; further studies are now needed to confirm this assumption. Finally, the global benefits of a CADe platform can only truly become fully appreciable only once the impact on the breadth of colonoscopy quality facets are assessed, including assessment of bowel preparation, mucosal surface area observed, polyp size and completeness of resection, as well as polyp characterization in addition to sole detection. Some of these may bear further potential cost savings, such as enhancing a polyp and discard strategy, although any true benefits remain currently unclear (49).

## CONCLUSION

The addition of CADe technology to colonoscopy is a cost-effective and dominant strategy (better outcomes at lower overall cost) for improving polyp detection in patients with a positive FIT in a Canadian universal health care system perspective when assessing LY and QALY over a patient’s lifetime.

## Data Availability

The data underlying this article is generated by an economic model subject to embargo for 18 months after publication. Once the embargo expires, data will be shared on reasonable request for research purposes.

## Abbreviations

ADR: adenoma detection rate
AI: artificial intelligence
AMR: adenoma miss rate
APC: adenoma per colonoscopy
CADe: computer-aided detection
CRC: colorectal cancer
ICER: incremental cost-effectiveness ratio
IRR: incidence risk ratio
LY: life years
QALY: quality-adjusted life year
WTP: willingness-to-pay
